# Characterization and toxicological potential of *Alternaria alternata* associated with post-harvest fruit rot of *Prunus avium* in China

**DOI:** 10.3389/fmicb.2024.1273076

**Published:** 2024-02-06

**Authors:** Tanvir Ahmad, Fuguo Xing, Changyu Cao, Yang Liu

**Affiliations:** ^1^School of Food Science and Engineering, Foshan University, National Technical Center (Foshan) for Quality Control of Famous and Special Agricultural Products (CAQS-GAP-KZZX043), Guangdong Key Laboratory of Food Intelligent Manufacturing, Foshan, Guangdong, China; ^2^Key Laboratory of Agro-Products Quality and Safety Control in Storage and Transport Process, Ministry of Agriculture and Rural Affairs, Institute of Food Science and Technology, Chinese Academy of Agricultural Sciences, Beijing, China; ^3^School of Life Sciences and Engineering, Foshan University, Foshan, Guangdong, China

**Keywords:** *Alternaria alternata*, *Prunus avium*, tenuazonic acid, alternariol, morphology, phylogenetics, physiology, pathogenicity

## Abstract

Post-harvest fruit rot caused by *Alternaria* species is one of the most important threats to the fruit industry. Post-harvest rot on sweet cherry (*Prunus avium*) fruit was observed in the fruit markets of the Haidian district of Beijing, China. The fungal isolates obtained from the infected sweet cherry fruits matched the descriptions of *Alternaria alternata* based on the morphology and multi-gene (ITS, endo-PG, and Alta1) sequence analysis. Pathogenicity tests indicated that ACT-3 was the most virulent isolate, exhibiting typical post-harvest fruit rot symptoms. Physiological studies revealed that the optimal conditions for the growth of ACT-3 were temperature of 28°C, water activity of 0.999, and pH of 8 with 87, 85, and 86 mm radial growth of ACT-3 on a potato dextrose agar (PDA) medium, respectively, at 12 days post-inoculation (dpi). Moreover, the fungus showed the highest growth on a Martin agar medium (MAM) modified (85 mm) and a PDA medium (84 mm) at 12 dpi. The proliferation of the fungus was visualized inside the fruit tissues by confocal and scanning electron microscope (SEM), revealing the invasion and destruction of fruit tissues. *Alternaria* mycotoxins, tenuazonic acid (TeA), and alternariol (AOH) were detected in five representative isolates by HPLC analysis. The highest concentrations of TeA (313 μg/mL) and AOH (8.9 μg/mL) were observed in ACT-6 and ACT-3 isolates, respectively. This study is the first to present a detailed report on the characteristics and proliferation of *A. alternata* associated with sweet cherry fruit rot and the detection of toxic metabolites.

## 1 Introduction

Sweet cherry (*Prunus avium*) is a highly valued and nutritionally important fruit with relatively low caloric content and a high vitamin C content, fiber, potassium, carotenoids, and polyphenols, which have antioxidant properties (McCune et al., 2010; Kelley et al., 2018; Ricardo-Rodrigues et al., 2023). According to FAO, sweet cherry production in China was recorded as 35,746 tons (FAOSTAT, 2021) and is expected to increase in the coming years.

Sweet cherry is susceptible to post-harvest rot caused by *Alternaria* species that affects the shelf life and quality of the fruit (Zhu and Xiao, 2015; Qiao et al., 2018). Several *Alternaria* species have been identified based on the morphological characteristics causing pre- and post-harvest diseases on a wide host range. However, often, there remains confusion and controversy about the taxonomy of *Alternaria* species due to overlapping morphological characteristics. Different molecular tools have been used to study the taxonomic status of several *Alternaria* species, and variable results were obtained (Simmons, 1992; Andersen et al., 2002).

The molecular study has an advantage over morphometric characterization for being more accurate. The combined study of physiological, morphological, and molecular characterization has provided comparatively reliable criteria to classify *Alternaria* species into specific groups within the genus *Alternaria* (Chen and Zhang, 1997). *Alternaria* is the most common genus of necrotrophic pathogens that cause post-harvest spoilage of various fruit crops in China (Wang et al., 2008, 2009; Cao et al., 2013; Liu et al., 2019).

*Alternaria* species cause significant loss at both pre- and post-harvest stages (Zhao and Liu, 2012; Lawrence et al., 2014; Troncoso-Rojas and Tiznado-Hernández, 2014; Woudenberg et al., 2015). They can even grow at low temperatures during storage (López et al., 2016). Therefore, different physiological parameters, including temperature, water activity, pH, and substrate, directly or indirectly influence the development of mycelial mass, sporulation, and its virulence. A systematic physiological study is required to understand the nature of the pathogen and devise its control strategies (Rotem, 1994; Troncoso-Rojas and Tiznado-Hernández, 2014).

Several *Alternaria* species can produce various types of toxic secondary metabolites or mycotoxins during their optimal growth and cause severe diseases in economically important crops (Meena et al., 2017; Meena and Samal, 2019). *Alternaria* mycotoxins are highly toxic substances commonly found in fruits, vegetables, and cereals worldwide. These mycotoxins are mainly divided into five groups based on their structure formation: (1) tetramic acid derivatives (TeA and isotenuazonic acid), (2) dibenzopyrone derivatives [AOH, altenuene (ALT), and alternariol monomethyl ether (AME)], (3) perylene quinone derivatives (atertoxins I, II, and III), (4) aminopentol esters, such as *A. alternata* f. sp. *lycopersici* (AAL) toxins, and (5) miscellaneous structures [tentoxin (TEN)]. Among these groups, TeA and AOH are predominant and occur in the highest concentrations in various food items (Lou et al., 2013; Bertuzzi et al., 2021; Chen et al., 2021). In terms of toxicity, TeA, AOH, and AME are the most potent *Alternaria* mycotoxins and cause cytotoxic, mutagenic, genotoxic, and carcinogenic effects in both humans and animals (Escrivá et al., 2017; Awuchi et al., 2021).

*Alternaria alternata and Alternaria tenuissima* in central Chile (Cancino et al., 2023) and *Alternaria arborescens* species complex (AASC) and *A. alternata* in Italy (Waqas et al., 2023) have been identified as the causal agents of black rot disease in sweet cherries based on the morphological and molecular analyses. These causal agents significantly contribute to yield losses. Although sweet cherry is an important fruit in China and is susceptible to post-harvest rotting by *Alternaria*, the detailed characteristics and multi-gene phylogeny of *Alternaria* species associated with sweet cherry fruit have not been described previously.

The objectives of this study were to identify *Alternaria* species associated with post-harvest fruit rot of sweet cherry using the morphological and molecular phylogenetic approaches and confirm their pathogenicity test to fulfill Koch’s postulates. The physiological characterization of *A. alternata* and its proliferation in host tissues were analyzed to determine the pathogenic nature and assay the infection in the sweet cherry fruit. This study also investigates the natural occurrence of *Alternaria* mycotoxins in the representative isolates of *A. alternata*. Understanding the pathogenic nature causing fruit rot disease on sweet cherry is critical for the implementation of disease management strategies to control post-harvest losses of sweet cherry fruit.

## 2 Materials and methods

### 2.1 Isolation

**Figure 1:**
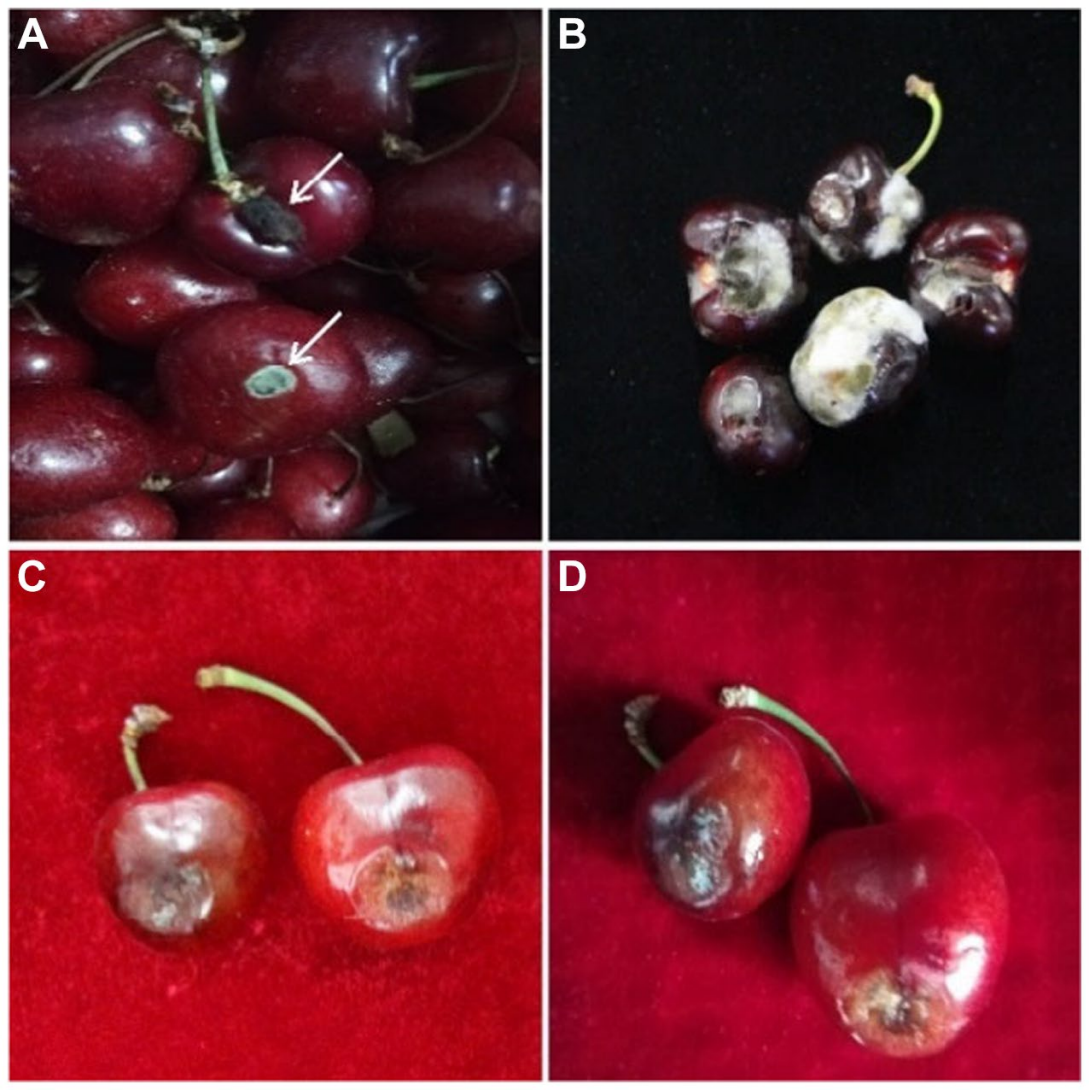
Disease symptoms of post-harvest fruit rot of sweet cherry disease; **(A,B)** samples collected from open boxes and **(C,D)** samples collected from packed boxes.

In June 2019, a survey was conducted at the local fruit markets of the Haidian district, Beijing, China. Post-harvest fruit rot of sweet cherry was observed in the market with an approximately 30% disease incidence (Figure 1). The samples were randomly collected from opened and packed boxes of sweet cherry fruits based on fruit rot symptoms and stored at 4°C. Infected tissues of each sweet cherry fruit sample were excised into small pieces (2–3 mm) with the help of a sterilized sharp scalpel, surface-disinfected with 1% sodium hypochlorite (NaOCl) for 60 s, and rinsed thrice with sterilized distilled water (SDW). To induce sporulation, these pieces were plated on a potato dextrose agar (PDA) medium and incubated at 28°C with 70% relative humidity (RH). Purified cultures were obtained through a single spore culture method on a PDA medium (Chen et al., 2018; Ahmad et al., 2019). All the isolates (*n* = 21) were preserved in 30% glycerol at −20°C.

### 2.2 Morphological characterization

To study the morphological characteristics of the pathogen, the 7-day-old fungal culture of each isolate was observed under a light microscope Olympus BX61. The data were recorded using CellSens Dimension (Olympus) software. The color and descriptions of conidia and mycelium were made by using the color chart of Rayner and the standard identification manual (Rayner, 1970; Simmons, 2007). The shape and size of 30 conidia per-isolate were observed and recorded. Furthermore, SEM analysis was carried out for ultrastructural observation. For sample preparation, the fungal mycelia were rinsed twice with phosphate buffer (pH 7) and fixed with 2.5% glutaraldehyde buffer (pH 7) for 24 h. Then, the sample was rinsed three times with 10 mM phosphate buffer and fixed for 2 h in 1% osmium tetroxide. Subsequently, each sample was subjected to gradient dehydration with 30, 50, 60, 70, 80, 90, 95, and 100% ethanol solution sequentially for 15 min. The samples were dried on a Leica EM CPD030 automated dryer (Leica Microsystems Inc., Germany) and fixed by a carbon tape on the sample stage of SEM. The gold coating of samples was done by ion sputtering “HITACHI MC1000.” The morphology was observed using a HITACHI SU8010 scanning electron microscope (Hitachi High-Technologies Corporation. Tokyo, Japan).

### 2.3 DNA extraction, PCR amplification, and sequencing

Genomic DNA was extracted from a 7-day-old culture of each isolate using a Prep-Man Ultra kit (Thermo Fisher ™) according to the manufacturer’s instructions. The sections of internal transcribed spacer (ITS), the endopolygalacturonase (*endo-PG*), and the major allergen (*Alt a1*) genes were amplified by PCR using the universal and specific primers: ITS1 (5’-CTTGGTCATTTAGAGGAAGTAA-3′), ITS4 (5’-TCCTCCGCTTATTGATATGC-3′) (White et al., 1990), PG3(5’-TACCATGGTTCTTTCCGA-3′), PG2b (5’-GAGAATTCRCARTCRTCYTGRTT-3′) (Andrew et al., 2009), Alt-for (5’-ATGCAGTTCACCACCATCGC-3′), and Alt-rev (5’-ACGAGGGTGAYGTAGGCGTC-3′) (Hong et al., 2005). The PCR conditions were as follows: an initial step of DNA denaturation at 95°C for 5 min, followed by 35 cycles of 30 s at 95°C, 30 s at 58°C, and 30 s at 72°C, with a final extension step at 72°C for 7 min. The PCR product was viewed on 1% agarose gel stained with ethidium bromide. The concentration of DNA was measured on a Nano-Drop (ND-1000) spectrophotometer, and then, sequencing was carried out at Shanghai Personal Biotechnology Co., Ltd., China. The sequencing method was Sanger dideoxy sequencing. All obtained sequences were cleaned and aligned using Bio Edit software (version 7.0.4), and five representative isolates were submitted to GenBank, NCBI, based on BLAST similarity analysis with deposited sequences.

### 2.4 Phylogenetic analysis

To unveil the phylogenetic relationships and taxonomic distinctions of *Alternaria* species, analyses were conducted using genetic markers recommended in the recent bibliography of *Alternaria* genus. Multiple sequence alignments of genes were concatenated by using ClustalX (Thompson et al., 2003) and adjusted manually. The phylogenetic analysis of ITS (522 bp), endo-PG (448 bp), and Alta1 (473 bp) gene regions in the present study, along with other *Alternaria* species previously deposited in GenBank (Supplementary Table S1), was conducted using Bayesian inference (BI) and maximum-likelihood (ML) analysis with MrBayes and RAxML software, respectively. The best-fit model of DNA evolution for the dataset in both BI and ML analyses was determined by jModelTest software (Darriba et al., 2012) using the Akaike information criterion (AIC). Maximum-likelihood (ML) analysis was performed using RAxML (Stamatakis, 2014) through RAxMLGUI v. 1.5b1 (Silvestro and Michalak, 2012). The analysis employed mixed models of evolution settings within the program, and bootstrap support was acquired through 1,000 replicates. Bayesian inference (BI) analysis was carried out using MrBayes software (Huelsenbeck and Ronquist, 2005). The objective was to estimate posterior probabilities (Huelsenbeck and Rannala, 2004) through Markov Chain Monte Carlo sampling (MCMC). Two parallel runs were executed with default settings, and six simultaneous Markov chains were run for 1,000,000 generations. The trees were sampled every 100th generation (resulting in 100,000 total trees). The initial 2,000 trees, representing the burn-in phase, were discarded. The remaining 8,000 trees were utilized to compute posterior probability values in the majority rule consensus tree.

### 2.5 Pathogenicity test

Detached medium-sized sweet cherry (Lapins cv.) fruits were selected and dipped into 1% NaOCl solution for 60 s and later rinsed three times with sterilized water for surface disinfection. Fruits were punctured with the help of a sterilized needle to make 4 mm × 2 mm [depth × width] wounds approximately, and 10 μL spore suspension (10^7^/ml) was deposited into each hole. Control fruits were treated with 10 μL sterilized distilled water only. All fruits were placed in 6-well culture cluster flat-bottom plates (costar 3,516) and covered with a zipper bag (240 × 170 mm) to maintain humidity. Samples were incubated for 7 days at 28°C. The disease incidence (%) was assessed by following the formula of Vincent (1947),


$$\text{Disease incidence}\;\left(\%\right)=\frac{\text{No}.\;\text{of infected fruits}}{\text{Total}\;\text{no}.\;\text{of inoculated fruits}}\times 100.$$

The virulence of the isolates was evaluated by measuring the diameter of the lesions on sweet cherry fruits. A five-point rating scale was used as described by Pryor and Michailides (2002) with slight modification: 0 = no lesion, 1 = lesions <1 mm in diameter, 2 = lesions 1 to 6 mm in diameter, 3 = lesions 6 to 12 mm in diameter, and 4 = >12 mm in diameter. The whole experiment was carried out three times under the same conditions.

### 2.6 Physiological characterization

#### 2.6.1 Preparation of spore suspension

Among all the isolates, the most dominant and virulent isolate ACT-3 based on the pathogenicity test was selected for detailed physiological study. The fresh conidial spore suspension was prepared using a 7-day-old culture of ACT-3 on a PDA medium. Spores were harvested in 10 mL sterilized distilled water amended with tween-80 (0.1%, v/v) by rubbing with the help of a sterilized loop. The concentration of spore suspension was maintained at 10^7^ spores/mL by using a hemocytometer and stored at 4°C.

#### 2.6.2 Effect of temperature and pH on fungal growth

To study the effect of temperature, 10 μL spore suspension (10^7^ spores/mL) was inoculated on PDA plates and incubated at 6, 12, 18, 25, 28, 31, 36, and 40°C at a 24-h dark photoperiod. For pH study, the PDA medium was adjusted to six different pH levels: 6, 7, 8, 9, 10, and 12 by adding HCL or NaOH and checked by a pH meter (Seven-Compact TM S210). Fungal mycelial diameter (mm) was measured at 3, 7, and 12 dpi.

#### 2.6.3 Effect of water activity (a_w_) and growth media

The effect of water activity (a_w_) on fungal mycelium was examined using the method described by Yin et al. (2022) with slight modifications. The a_w_ of the PDA medium was maintained at 0.99, 0.98, 0.97, 0.96, 0.95, 0.94, 0.92, and 0.91 by adding different amounts (v/v) of glycerol (HOCH_2_CHOHCH_2_OH) (Xilong Scientific Co., Ltd. Guangdong, China), and a_w_ was checked three times at 6-h intervals by a Water Activity Meter (AQUA-LAB Dew point a_w_ meter 4TE, PYNN160703) before inoculation. Additionally, the prepared PDA plates were also stored to check the deviation of a_w_ at the end of the experiment. Nine growth media (Supplementary Table S2) were prepared according to the manufacturer’s instructions. In addition, 20 mL of each medium was poured into Petri plates and kept at room temperature for solidification. All the plates were inoculated with 10 μL spore suspension (10^7^ spores/ml) and incubated at 28°C in 24-h dark and 24-h light (LED-05). Fungal mycelial diameter (mm) was measured at 3, 7, and 12 dpi.

The experiment was repeated three times under the same conditions, and fungal characteristics including color and growth pattern were recorded by visual observations and compared with the color chart given by Rayner (1970).

### 2.7 Proliferation of fungal hyphae in cherry fruit

Medium-sized fresh sweet cherry fruits were artificially inoculated with 10 μL inoculum following the method of Lang (2003) and incubated following the same method described above in section 4.5. At 7 dpi, approximately 0.25 grams (g) of rotten tissues were excised from the infected fruit. For the confocal study, fungal and sweet cherry fruit tissues were visualized by staining with 10 μg/mL wheat germ agglutinin-Alexa Flour 488 (WGA-AF488) conjugate (Thermo Fisher Scientific, W11261) and propidium iodide (PI) (Sigma-Aldrich, P4170), respectively (Yang et al., 2017; Redkar et al., 2018), with slight modifications. The harvested tissues from inoculated fruit at 7 dpi were gradient eluted in 40, 60, 80, and 100% ethanol each for 10 min and replaced with 100% ethanol for 24 h to undergo bleaching and complete removal of natural pigments of fruit tissues. Later, the ethanol was replaced with 5% KOH and incubated for 2–3 min to make the tissues more transparent. The samples were washed five times in PBS (pH 7) for the efficient WGA-AF488 staining. Then, the samples were completely immersed in the staining solution (WGA-AF488 and PI in PBS) in microcentrifuge tubes. Confocal images were taken with a Leica TCS-SP8 confocal microscope (Leica^1^) with the emission spectra of 488/500–520 nm for WGA-AF488 and 561/590–640 nm for PI. Three replicates were evaluated for each treatment. For SEM, the excised tissues were treated the same way as described above in the “Morphological characterization”section.

### 2.8 Toxicological potential of *Alternaria* mycotoxins

#### 2.8.1 Sample extraction

Extractions of mycotoxins from fungal isolates were carried out according to the method of Meena et al. (2017) with a slight modification. Two agar plugs were cut from the center of a 7-day-old culture of each representative isolate, inoculated with a 200-mL potato dextrose broth (PDB), and incubated for 15 days at 28°C and 10 rpm. Cultures were filtered through 5-μm microporous filter papers (50 mm) by the SHZ (III) vacuum pump (China). Subsequently, an equal amount of methanol was added to the culture elute/filtrate and kept at 4°C for 24 h. Then, the culture filtrate was precipitated and concentrated under a gentle stream of nitrogen at 45°C. An equal volume of ethyl acetate was added to the obtained extract and mixed properly in a separatory funnel. Two phases were obtained: aqueous and organic phases. The aqueous layer was separated and extracted with ethyl acetate. The ethyl acetate extract was concentrated at 45°C under a gentle stream of nitrogen and dissolved into an equal volume of methanol.

#### 2.8.2 Preparation of the standard

TeA and AOH standards were purchased from Pribolab, Beijing, China and used in a crystallized form to prepare the standard. The stock solutions (1,000 μg/mL) of toxins were prepared in HPLC grade methanol (w/v) and stored at −18°C in dark conditions. Stock solutions of toxins were diluted with HPLC grade methanol to maintain different concentrations.

#### 2.8.3 HPLC analysis for the detection of TeA and AOH

For HPLC analysis, the samples were separated using an Agilent-5 TC-C 18(2) (250 mm long×4.6 mm, 5.0 μm particle size) column (product No: PN588925-902, SN554821), which was connected to the guard column. The Waters series system (Waters Corporation, Alliance, USA) has a multiple λ fluorescence detector (2,475 FLD) and a Waters 2,690 Separation module system controller. Then, a 10-μL sample was injected. The column was thermostatically controlled at 30°C, and each sample was run for 10 min. The flow rate was 1 mL/min, and the mobile phase consisted of 75% HPLC grade methanol (solvent A) and 25% of an aqueous solution (solvent B) of 0.1 M phosphate buffer. The HPLC was run in an isocratic mode, and the detection was monitored at the range of 200–440 nm. To determine the linear relationship response, different concentrations of standard TeA and AOH were analyzed by using HPLC. To ensure the reliability and sensitivity of the HPLC method, the limit of detection (LOD) and the limit of quantification (LOQ) were determined as per signal-to-noise ratio according to the guidelines of International Council on Harmonization (ICH) (Guideline, 2005). For recovery and precision validation, blank culture medium samples were spiked with TeA and AOH standards at various known concentrations ranging from 3 to 9 μg/mL.

### 2.9 Statistical analysis

The data were tested by a Shapiro–Wilk test to assess data normality, and data were found to be normally distributed prior to statistical analysis. The data were subjected to a one-way analysis of variance (ANOVA), and the difference between treatment means was separated using the least significant difference (LSD) test at *p* ≤ 0.05 using Statistix statistical package (ver. 8.1). The results are presented as the mean ± standard error (SE), mean ± standard deviation (SD), and % relative standard deviation (% RSD), as estimated from Microsoft Excel (Microsoft 365, Excel Ver. 2,306).

## 3 Results

### 3.1 Isolation and morphological characterization

**Figure 2:**
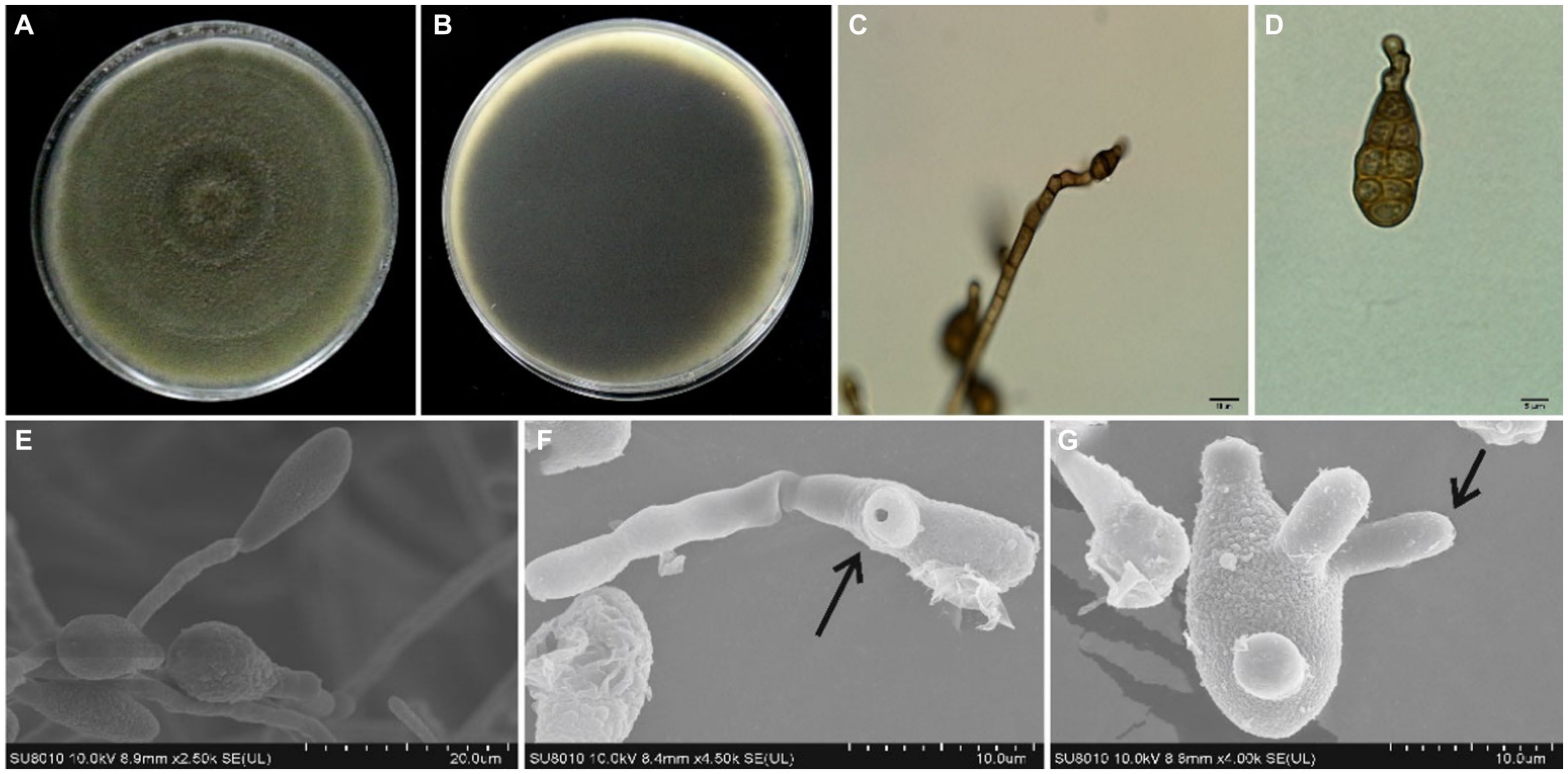
Morphological features of *A. alternata* isolated from sweet cherry fruit, **(A)** the front and **(B)** reverse side of fungal colony on PDA, **(C)** conidiophore, **(D)** conidium, **(E)** production of conidium, **(F)** germ tube emerging from the germinated spore, and **(G)** production of germ tubes.

A total of 21 *Alternaria* fungal isolates were obtained from the infected sweet cherry fruit tissues. Initially, all these isolates were identified as *A. alternata* based on morphology characteristics and BLAST analysis. Subsequently, five representative isolates were selected for further studies. The isolates grew well (8 mm per day at 28°C) on the PDA medium and showed the same morphological appearance. All the isolates formed circular and dark olive green colonies with gray to white aerial mycelia on the PDA medium at 28°C after 7 days. The five isolates were selected as representatives and were further studied by using a light microscope. All the representative isolates produced primary (7–13 conidia per chain) and secondary (1–5 conidia per chain) conidial chains. The conidial chains were usually branched in a sympodial way due to the extensions of secondary conidiophores from the basal conidial cells. Conidia were light to dark brown in color, irregular, and ovate to obclavate in shape. Conidia usually showed 0–3 longitudinal and 1 to 9 transverse septations (Figure 2). SEM analysis of isolates demonstrated that the conidiophores were short, straight, or slightly curved. SEM analysis indicated that the conidia produced the germ tubes. The size of conidia from all isolates varied from 9.34 to 36.4 μm in length and 4.9 to 12.01 μm in width. The beak length of conidia varied from 3.3 to 9.1 μm (Table 1). Based on these characteristics, all the isolates were primarily identified as *A. alternata*.

**Table 1:** Measurements of conidia of representative *A. alternata* isolates.^a^

Isolate	Length (μm)	Width (μm)	Length/width (μm)	Beak length (μm)
ACT-2	24.7 ± 7.6 (9.3–36.4)	7.0 ± 1.9 (4.5–10.6)	3.8 ± 1.2 (1.4–5.9)	5.9 ± 1.4 (3.3–8.0)
ACT-3	20.6 ± 5.9 (10.1–32.2)	7.4 ± 2.5 (4.1–13.1)	1.1 ± 1.4 (1.3–7.5)	7.1 ± 0.9 (5.6–9.1)
ACT-4	29.4 ± 5.7 (18.0–36.0)	8.0 ± 1.5 (4.6–11.3)	1.0 ± 1.3 (1.9–7.4)	5.2 ± 0.8 (3.8–7.3)
ACT-5	24.0 ± 6.6 (12.3–34.1)	8.3 ± 1.3 (5.2–10.2)	3.1 ± 1.1 (1.3–5.6)	5.2 ± 0.8 (4.0–6.7)
ACT-6	21.1 ± 6.1 (11.4–32.2)	8.4 ± 1.6 (5.4–12.3)	2.6 ± 0.8 (1.1–4.9)	5.3 ± 0.8 (3.6–6.7)

^a^Values are an average of 30 replications ± standard deviation (SD) (minimum value–maximum value).

### 3.2 Molecular sequencing and phylogenetic analysis

**Figure 3:**
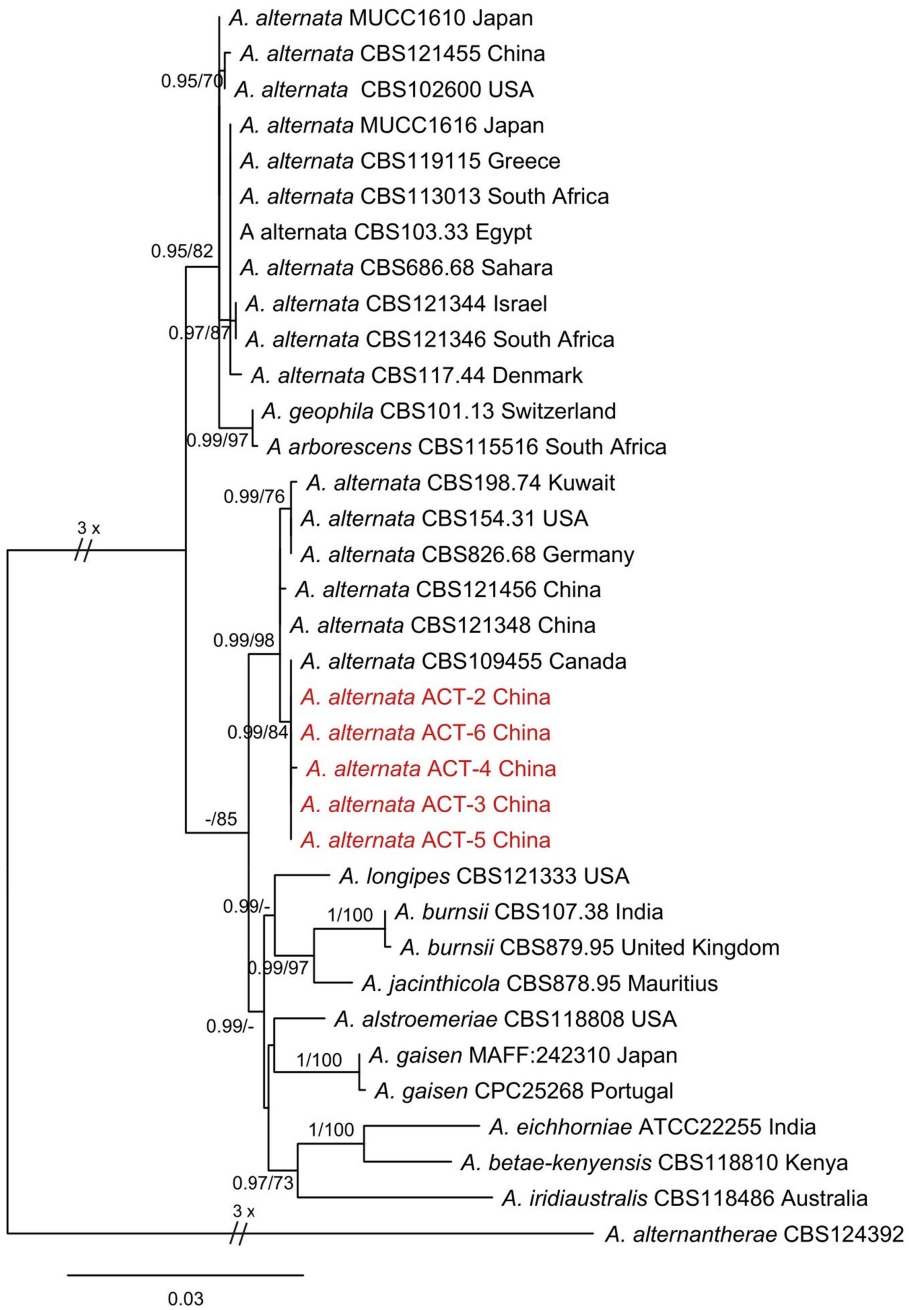
Phylogenetic tree was constructed through maximum likelihood analysis (RAxML) based on combined *ITS*, *endo-PG*, and *ALTa1* sequences. *Alternaria alternantherae* CBS124392 was used as an out-group. The numbers above the branches represent bootstrap percentages **(right)** and Bayesian posterior probabilities **(left)**. The support values of bootstrap are ≥70%, and Bayesian posterior probabilities are ≥0.95. The sequences of *Alternaria* species obtained in this study are indicated in red color.

The evolutionary status of representative isolates was studied by amplifying three different gene fragments: ITS, endo-PG, and Alta1. The BLAST analysis of ITS, endo-PG, and Alta1 sequence data indicated that the isolates showed 99% similarity with *A. alternata* species in the NCBI, GenBank database. Phylogenetic analysis of the concatenated sequences of ITS, endo-PG, and Alta1 genes resulted in a phylogenetic tree that well separated *Alternaria* species in different clades with high bootstrap (≥70%) and Bayesian posterior probability (≥0.95) values. All the isolates from this study clustered together in a well-supported clade with reference *A. alternata* isolates from Human arm tissue (Canada)*, Sanguisorba officinalis* (China)*, Platycodon grandiflorus* (China)*, Lolium* sp. (Germany), *Staphylea trifolia* (USA), and soil (Kuwait) substrates with 98% bootstrap and 0.99 posterior probabilities values (Figure 3). Two separate *A. alternata* clades were observed in phylogenetic analysis, and divergence into these clades may be indicative of different substrates. Therefore, based on morphological characteristics, molecular sequencing, and phylogenetic analysis, the species infecting sweet cherry fruit was identified as *A. alternata*.

### 3.3 Pathogenicity

**Figure 4:**
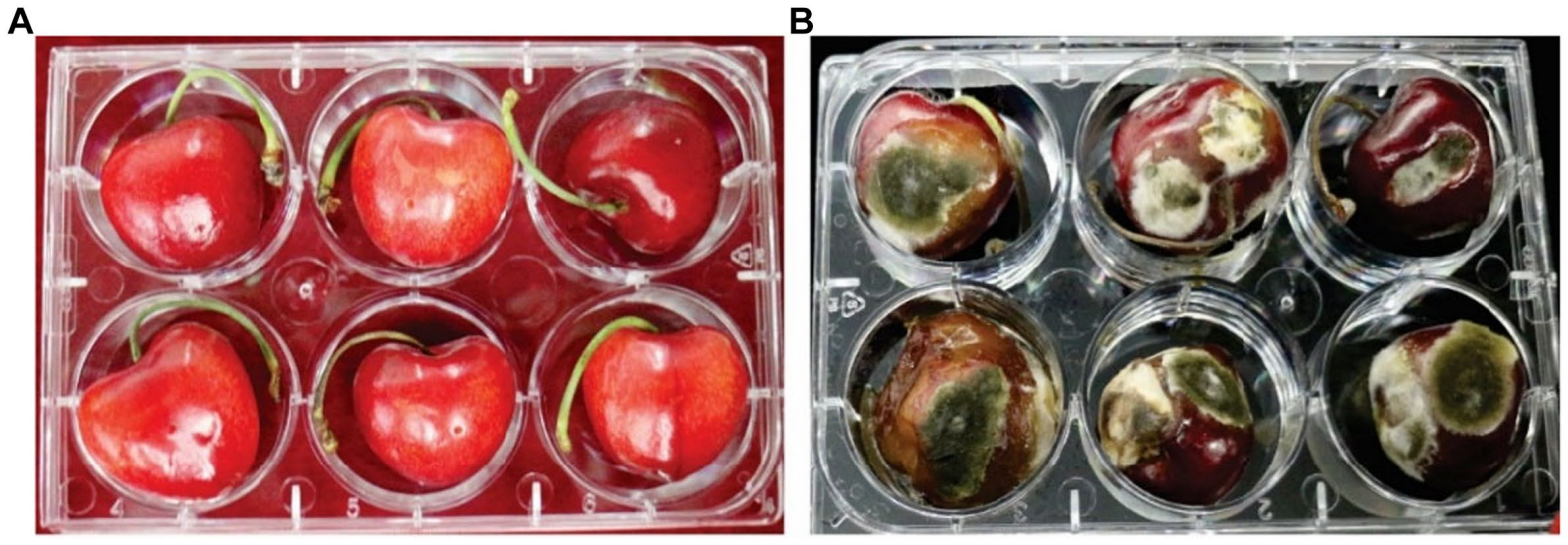
Pathogenicity of *A. alternata* on cherry fruit. **(A)** Control cherry fruit without symptoms and **(B)** inoculated cherry fruit exhibiting fruit rot symptoms at 28°C.

Irregular soft rot patches with fungal hyphae were observed around the inoculation site causing sweet cherry fruit rot at 7 dpi. These disease symptoms were similar to those observed on original samples collected from fruit markets. Control sweet cherry fruits remained asymptomatic (Figure 4). At 7 dpi, 72.2 to 94.4% of sweet cherry fruits developed symptoms. Isolate ACT-3 was the most virulent and produced a 10.2-mm-diameter lesion around the inoculation point (Table 2). Fungal isolates produced up to 70% decayed soft lesions on the sweet cherry fruit surface. The re-isolated pathogen from the sweet cherry fruit resembled the original isolate based on the morphological and molecular characterization.

**Table 2:** Pathogenicity of *A. alternata* isolates on sweet cherry fruit at 7 dpi.

Isolate	DI (%)^a^	Lesion diameter (mm)^a^
ACT-2	83.3 ± 2.6 ab	9.2 ± 1.3 b
ACT-3	94.4 ± 1.0 a	10.2 ± 1.7 a
ACT-4	85.1 ± 0.5 ab	9.0 ± 0.6 b
ACT-5	79.6 ± 0.5 bc	8.6 ± 1.0 b
ACT-6	72.2 ± 0.1 c	8.5 ± 0.8 b
Control	0.0 ± 0.0 d	0.0 ± 0.0 c

^a^The values across the columns with the same letters are not significantly different from each other and are analyzed by the LSD test at *P* ≤ 0.05 after data were subjected to a one-way ANOVA. Values are the average of 18 replicates ± SD.

### 3.4 Physiological characterization

#### 3.4.1 Effect of temperature

**Figure 5:**
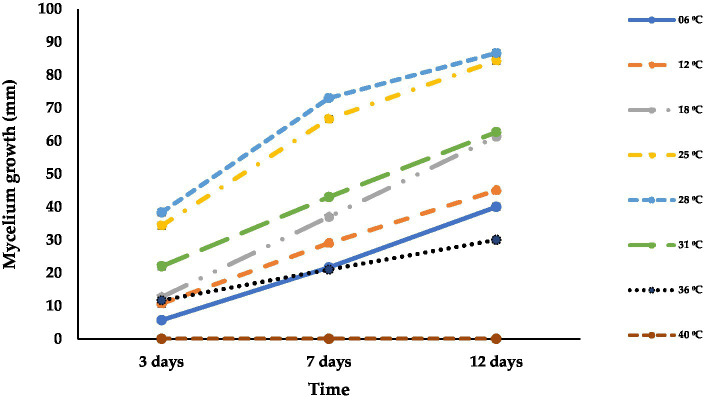
Effect of different temperatures (°C) on the mycelial growth of *A. alternata* isolate ACT-3. Bars indicate the standard error (SE) of mean values.

Isolate ACT-3 was able to grow at different temperatures ranging from 6^°^C to 36^°^C. ACT-3 grew well at 28°C (87 mm) followed by 25°C (84 mm), and the least growth was observed at 36°C (30 mm) at 12 dpi. At 40°C, ACT-3 failed to grow which appeared to be the thermal inactivation point of this isolate (Figure 5). Temperatures ranging from 25 to 28°C were found to be optimal for the growth and sporulation of *A. alternata.* The fungal colony showed green to light brown color with light gray to white aerial mycelium at 3 dpi at 25°C and 28°C; but at 12 dpi, the colony color turned into olive green to dark brown. The fungal colonies were generally light green to off white, olive green, and olive brown at 3, 7, and 12 dpi, respectively, at 6 and 12°C on the PDA medium (Supplementary Figure S1).

#### 3.4.2 Effect of pH

**Figure 6:**
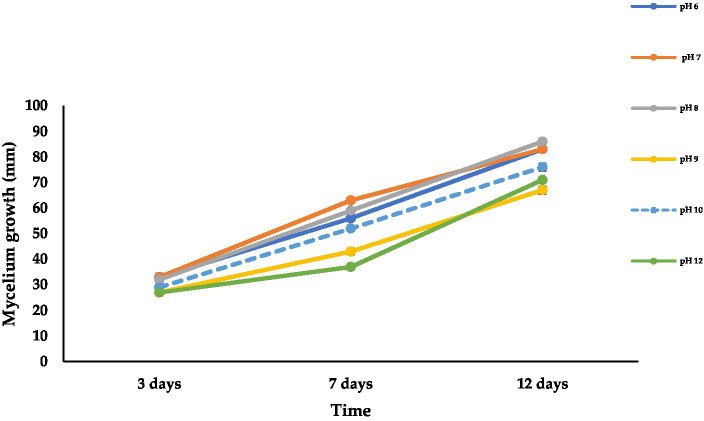
Effect of different pH levels on mycelial growth of *A. alternata* isolate ACT-3 on PDA. Bars indicate SE of mean values.

Data presented in Figure 6 reveal the differences in the colony growth of *A. alternata* at different pH levels. The mean radial colony growth varied from 67 to 86 mm at 12 dpi at all tested pH levels. The fungal mycelium grew well at all tested pH levels, and maximum radial growth (86 mm) was recorded at pH 8 followed by pH 6 to 7 (83 mm) and pH 10 (76 mm), while the lowest mycelial growth was recorded at pH 9 (67 mm) followed by pH 12 (71 mm) at 12 dpi on PDA at 28^°^C in 24 h darkness. The fungus showed light green to olive color, olive to light brown, and olive green to brown at 3, 7, and 12 dpi, respectively (Supplementary Figure S2).

#### 3.4.3 Effect of water activity and medium

**Figure 7:**
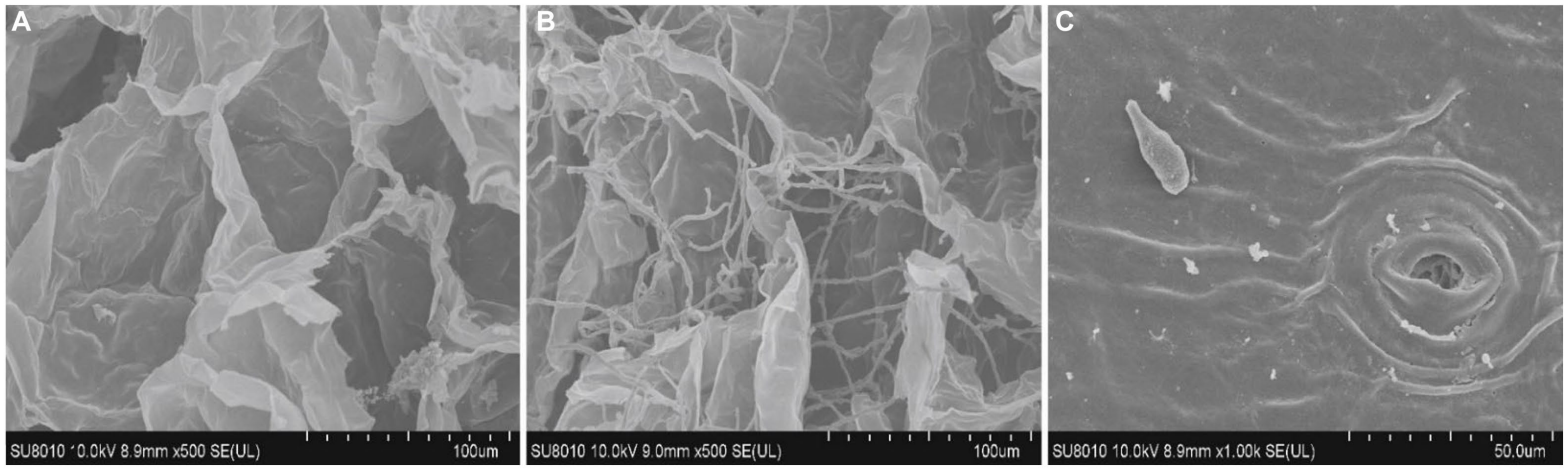
Proliferation of *A. alternata* hyphae in fruit tissues under scanning electron microscopy: **(A)** control; **(B)** treated with the fungus invaded by fungal hyphae; and **(C)** conidia production at the inner side of epidermal tissues. Scale bar; 100 μm **(A,B)** and 50 μm **(C)**.

**Figure 8:**
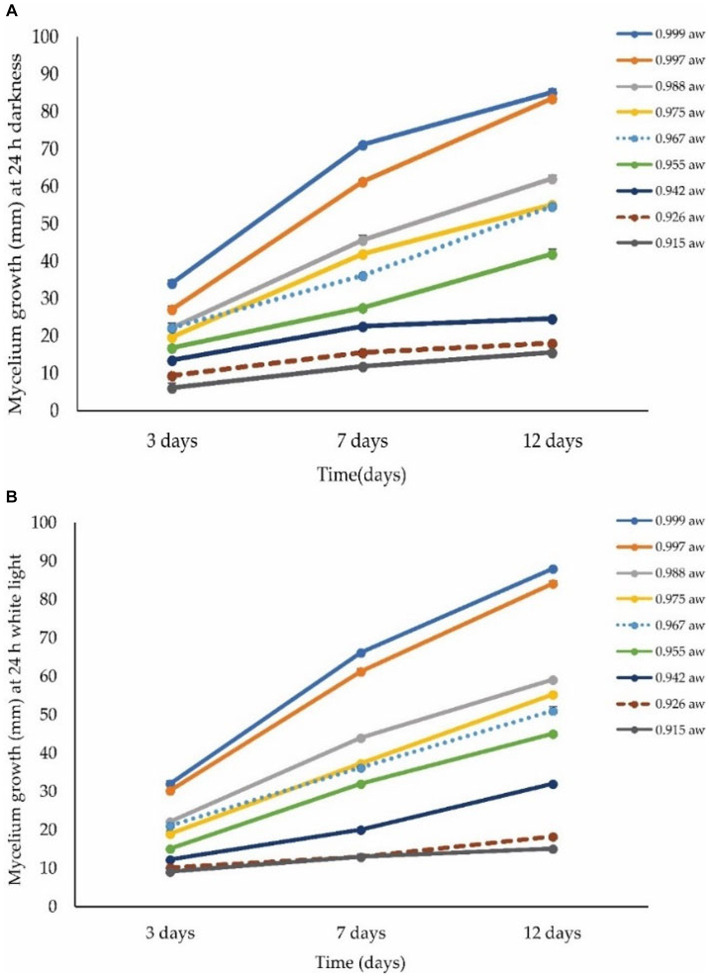
Effect of different levels of water activity (a_w_) on the growth of *A. alternata* isolate ACT-3. **(A)** Mycelial growth under 24-h darkness on different levels of a_w_. **(B)** Mycelial growth under 24-h light exposure (LED 5 integrated positive white light) on different levels of a_w_ on PDA medium. Bars indicate the SE of mean values.

Figure 7 shows the radial growth of *A. alternata* at different levels of a_w_ on the PDA medium. The optimum water activity for fungal growth was 0.999 a_w_ (85 mm) followed by 0.997 a_w_ (84 mm) and 0.988 a_w_ (62 mm), while the minimal condition for growth was 0.915 a_w_ (16 mm) followed by 0.926 a_w_ (18 mm) and 0.942 a_w_ (25 mm) on the PDA medium at 12 dpi at 28°C in 24-h darkness (Figure 8A). The optimal conditions for growth were observed at 0.999 a_w_ with a colony size of 88 mm, followed by 0.997 a_w_ (84 mm) and 0.988 a_w_ (55 mm). Conversely, the minimal conditions for growth were found at 0.915 a_w_ (15 mm), followed by 0.926 a_w_ (18 mm) and 0.942 a_w_ (32 mm) on the PDA medium after 12 dpi at 28°C under 24 h of light exposure (LED 5 integrated positive white light) (Figure 8B). The best levels of a_w_ ranged from 0.999 to 0.988 at which *A. alternata* showed the highest growth. The growth rate increased with increasing water activity. At 3, 7, and 9 dpi, the colony color at different levels of a_w_ was observed as light green to dark brown and off white to light creamy in 24-h dark and light conditions, respectively (Supplementary Figure S3). Growth of *A. alternata* isolates from the current study on different media is presented in Supplementary File S1.

### 3.5 Proliferation of *Alternaria alternata* hyphae in sweet cherry fruit tissues

**Figure 9:**
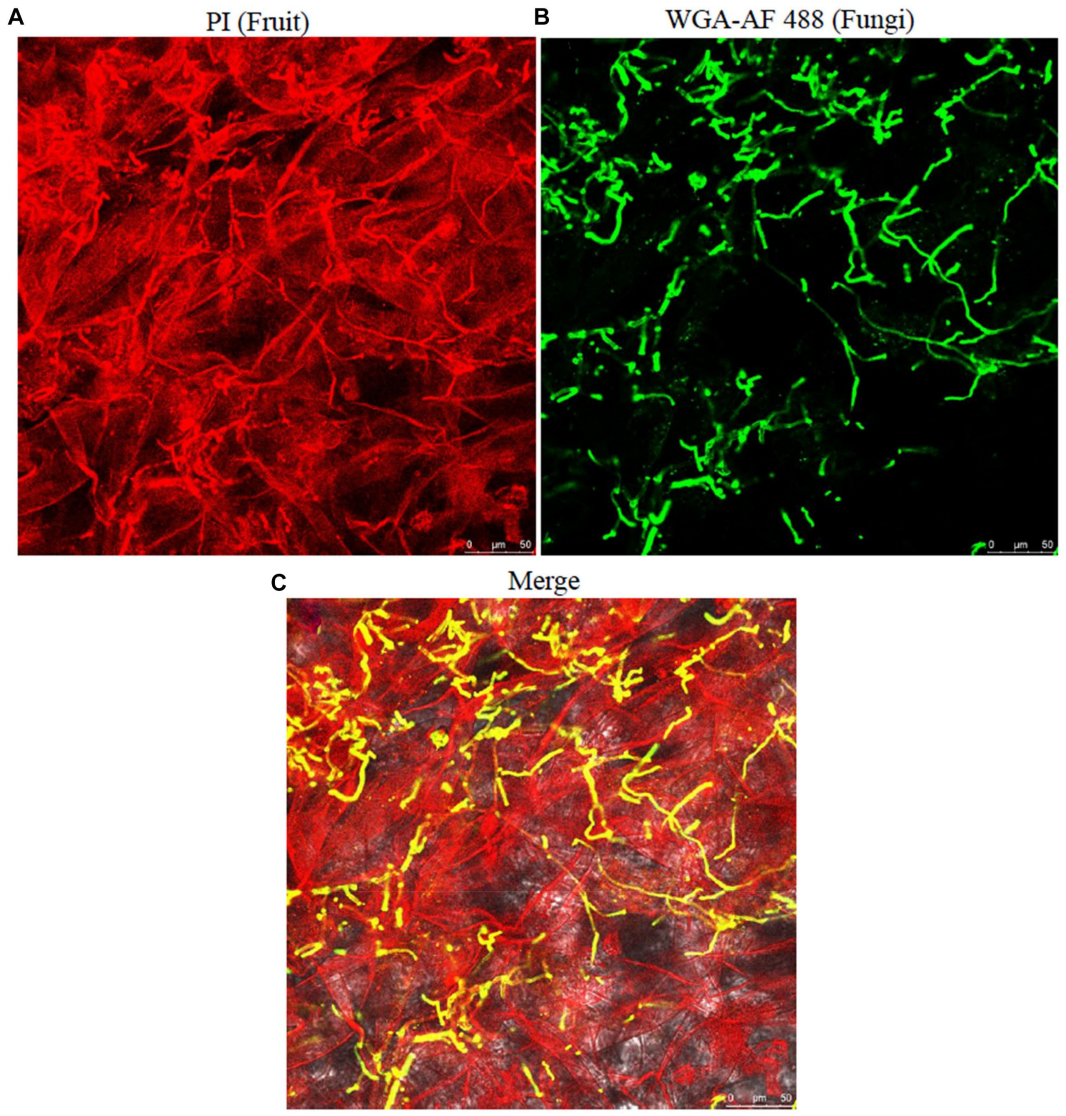
Proliferation and visualization of *A. alternata* infection in cherry fruit tissues. **(A)** fruit tissues; **(B)** fungal hyphae; **(C)** merged; fungal hyphae showing colonization and hyphal growth within the fruit tissues. Scale bar; 50 μm **(A–C)**.

To check the proliferation of the fungus in sweet cherry fruit tissues, the infected fruit tissues were examined by confocal microscopy. The fungal hyphae and fruit tissues were emitting green and red fluorescence, respectively. The fungal colonization started from inside the fruit cuticle and then entered the epidermal cells. The fungus then formed hyphal branches inside the fruit tissues. The results revealed that conidia and hyphae entered through the stomatal and epidermal cells and disrupted the morphology of fruit cells within 7 days (Figure 9). SEM analysis of the infected tissues revealed that *A. alternata* produced hyphae and conidia, which intermingled with fruit tissues and the inner surface of the epidermal tissues of the fruit, respectively. The conidia entered through the fruit cuticle and germinated rapidly inside the host without showing any initial symptoms. The fungal hyphae spread between the cells in irregular manners and then the fruit exhibited softening, and after that, the mycelium appeared on the fruit surface (Figure 7). Fungal hyphae of *A. alternata* showed direct proliferation within sweet cherry fruit and caused tissue rotting and disturbed the fruit cell morphology, ultimately causing spoilage of the fruit.

### 3.6 Detection of TeA and AOH

The representative isolates were analyzed for the mycotoxin profile. For each isolate, TeA and AOH production was assessed by HPLC analysis, which demonstrated that the TeA and AOH standard peaks were observed at retention times (RT) of 3.31 and 6.60 min, respectively. In tested samples, the retention times of TeA and AOH toxins were the same as the standards of TeA and AOH, confirming the presence of these mycotoxins. HPLC analysis of isolates is presented based on the same RT with standards (Supplementary Figure S6). The concentrations (μg/mL) of TeA and AOH were determined based on the peak area. The linear relationship between the detector response and different concentrations of TeA and AOH was obtained. The calibration curve equation of TeA (*y* = 5178.3x−12356) and AOH (*y* = 9748.5x–2642.1) was used to measure the concentrations of TeA and AOH from the culture medium, and coefficient *R*^2^ was greater than 0.99 for both standard curves. Both TeA and AOH concentrations showed a linear relationship with a chromatographic peak area. LOD and LOQ for TeA and AOH were calculated as 3.47 μg/mL and 10.52 μg/mL for TeA and 0.54 μg/mL and 1.65 μg/mL for AOH, respectively. The recovery of TeA varied between 84.55 and 92.96%, with a % RSD ranging from 4.53 to 9.93%. For AOH, the recovery ranged from 87.78 to 96.30%, with a % RSD varying between 2.40 and 5.80%. All tested isolates produced TeA and AOH except ACT-6, which did not produce AOH. ACT-6 isolate was found to produce a maximum concentration of TeA (313.47 ± 1.2 μg/mL), and ACT-2 isolate showed a maximum concentration of AOH (8.99 ± 0.2 μg/mL) in culture medium (Table 3).

**Table 3:** Concentrations of two toxins: tenuazonic acid (TeA) and alternariol (AOH) from representative *A. alternata* isolates.

Sr#	Isolates	TeA (μg/mL)	AOH(μg/mL)
1	ACT-2	293.96 ± 1.1	1.72 ± 0.1
2	ACT-3	80.04 ± 0.6	8.99 ± 0.2
3	ACT-4	204.53 ± 1.0	2.43 ± 0.1
4	ACT-5	50.58 ± 0.9	3.90 ± 0.1
5	ACT-6	313.47 ± 1.2	ND

Mean values ± SD of the average of three replicates of each isolate; ND, not detected.

## 4 Discussion

The current study presents a detailed approach to characterize *A. alternata* associated with the post-harvest fruit rot of sweet cherry in China based on morphology, multiple gene sequence comparisons, phylogeny, proliferation in host tissues, physiology, and mycotoxin production. In major stone fruit growing regions, the infection of *A. alternata* is very common in stone fruits after harvesting and during storage. The results of cultural and morphological characteristics of *A. alternata* were similar to the previous studies in which *A. alternata* caused post-harvest fruit rot of *Syzygium cumini* and citrus black spot (Wang et al., 2010; Ahmad et al., 2019). According to several previous reports, all isolates of *A. alternata* isolated from several hosts produced dark olive green colonies on the PDA medium with dark brown conidia at 25 ± 2°C in a dark environment (Chen et al., 2018; Ghuffar et al., 2018; Alam et al., 2019), and the size of the conidia was 12–30-μm long and 7.5–12 μm wide (Gur et al., 2017; Chen et al., 2018; Moosa et al., 2019). Previous studies reported similar results that the length of conidia varied from 0 to 10 μm (Kumar et al., 2012; Lin et al., 2015). The causal organism of sweet cherry rot was preliminarily identified as *A. alternata* based on their morphological features (Simmons, 2007).

However, an identification based only on the morphological features is not a reliable method as it can lead to misidentification. Therefore, for more accurate identification of the isolates at the species level, we used a polyphasic approach that was based not only on morphological characterization but also on DNA sequence comparisons, phylogeny, physiological parameters, and mycotoxin production. The multi-locus phylogenetic analysis revealed that all the isolates in this study were more closely related to *A. alternata* with high similarity. Phylogenetic analysis confirmed the presence of only a single species of *A. alternata*. A single gene region ITS is not enough to differentiate among *Alternaria* species, and the phylogenetic trees derived from more than one gene region were used to resolve species differentiation in the genus *Alternaria*. In several previous studies, the three gene regions selected in this study provided an accurate identification of *Alternaria* species (Andersen et al., 2009; El Gobashy et al., 2018; Ramires et al., 2018).

The pathogenicity test indicated that all isolates are pathogenic and caused post-harvest rot of sweet cherry fruit. Similar observations of symptoms and decayed tissues have been observed on different fruits (Liu et al., 2019). *A. alternata* and *A. arborescens* isolates induced typical symptoms of heart rot disease on artificially inoculated pomegranate fruit, and the rot extended to approximately half of the entire longitudinal section of the fruit (Aloi et al., 2021).

According to physiological studies, temperature, water activity, substrate or growth medium, and pH are the important factors that influence the growth of fungal pathogens (Azad et al., 2016; Reddy et al., 2019). In agreement with our findings, the optimum temperature and pH for *A. alternata* mycelial growth on the PDA medium were recorded as 25 + 2^°^C and 6.5, respectively (Singh et al., 2001). Ginoya and Gohel (2015) also reported that the optimal temperature for *A. alternata* growth is 28^°^C, and the results obtained by the current study confirm to this report. The maintenance of appropriate temperatures during storage and transportation can be pivotal in increasing shelf life of sweet cherries. Lower temperatures can play an important role in minimizing *A. alternata* infection (Wang et al., 2020). In confirmation of our research, the optimum growth of *A. alternata* isolates was observed on a growth medium having 6–8 pH (Hubballi et al., 2010). Reddy et al. (2019) reported that *A. alternata* grew moderate to optimum on all the tested media, but the PDA medium was the best growth medium. Similar results were also obtained in the present study. Fungal colonies were dark brown on the reverse side, while the colonies appeared olive to brown colored from the front side on the PDA medium. On MEA, the initial color of the colony was light greenish to brown and off white at the colony edges in a 24-h dark environment. The continuous light reduced the sporulation of *A. alternata*, and the colony appeared light brownish with a grayish to off white color supported by a previous study (Kiran et al., 2018).

Water activity is a very important factor in the growth of pathogenic fungi. According to a previous report, the optimal a_w_ was 0.97–0.99 at 25–28^°^C for *A. alternata*, which showed more than 70% growth while the lowest growth was observed at a 0.95 a_w_ level, and these results support our findings (Prendes et al., 2017). High a_w_ in stored cherries can accelerate microbial growth, including *Alternaria* fruit rot, compromising the fruit’s quality and shelf life. Controlling water activity through appropriate storage conditions can mitigate sweet cherry fruit rot caused by *A. alternata* and preserve overall post-harvest quality of sweet cherry fruit.

The confocal microscopy results showed that *Alternaria* sp. began to invade at approximately 4 h post-inoculation (hpi), and the mycelial branches formed at 6 hpi at several hyphal branches then expanded into the adjacent tissues at 12 hpi. At 20 hpi, the hyphae formed a multicellular network supported by the findings of Yang et al. (2017). Our results comply with the study by Dehpour et al. (2007) who reported that the conidia of *A. alternata* can move through stomata from the outer skin surface to inside the host parenchymatous tissues causing infection and can also directly penetrate fungal hyphae through fruit cuticle in the host parenchymatous tissues as observed by SEM analysis.

The hyphal branches of *A. alternata* expanded into the intracellular spaces of tissues. *Alternaria* species are known to produce mycotoxins while proliferating into host tissues causing cell injury and decay (Mao et al., 2023). *Alternaria* species produce a wide range of metabolites including mycotoxins at optimal growth conditions. The different metabolites, including TeA, AOH, cyclic tetrapeptide tentoxin (TEN), tetramic acids, dibenzopyrones, cyclic peptides, lactones, and quinones, have been reported from *Alternaria* species causing toxicity (Patriarca et al., 2007; Ostry, 2008; Lee et al., 2015). These mycotoxins are very common food contaminants in fruits including berries (Fan et al., 2016), pomegranate (Elhariry et al., 2016), apple and citrus (Gabriel et al., 2017; Gur et al., 2017), and vegetables (Solfrizzo et al., 2004). In this study, we have confirmed the presence of TeA and AOH in our isolates through HPLC analysis based on retention time. The detection of *Alternaria* mycotoxins through several analytical techniques, i.e., TLC, HPLC, and ESI MS/MS, has been reported in several previous studies (Rodríguez-Carrasco et al., 2016; Meena et al., 2017).

*Alternaria* toxins have been reported in figs, cereals, sunflower seeds, and vine crops. The isomers of *Alternaria* toxins were also detected in fresh fruits and olives (López et al., 2016). Our results also revealed that *A. alternata* isolates produced TeA in maximum amount as compared to AOH (Table 3). Chain (2011) has also stated that TeA has the highest occurrence and contamination in fruit products and their by-products. The toxicological potential of *A. alternata* isolates associated with heart rot disease of pomegranate fruit in the liquid culture medium was investigated and five mycotoxins extracted with different concentration levels. All isolates produced TeA which found prevalent mycotoxin in culture extract, while AOH, AME, TEN, and benzopyrone derivatives were produced by 70% of the isolates (Aloi et al., 2021). The findings of the present study are consistent with previous reports, indicating that *A. alternata* produced mycotoxins in the culture medium. *Alternaria* mycotoxins, such as TeA, AOH, TEN, and AME, have a broad host range, and they might act as virulence factors in fruit rot diseases (Wenderoth et al., 2019). The influence of post-harvest diseases and contamination of mycotoxins in fruits are an enraging threat in the agriculture and food security sector.

Characterization and toxigenic profile of sweet cherry fruit rot pathogen is very important to control the early stage infection and mycotoxins contamination. These research findings can be helpful in identifying the causal agent on time and controlling the disease stage of infection, which can minimize the health risks of sweet cherry consumers. This study illustrates the presence of *A. alternata* in sweet cherry fruit that are consumed daily. This pathogen is also a high producer of TeA and AOH mycotoxins in the culture medium. However, a few steps including early fungal pathogen detection, its characterization, and toxigenic potential can be helpful to avoid human exposure to *A. alternata*, and we can also enhance fruit shelf life for longer periods of time. These steps are the implementation for the critical control in places where sweet cherry fruit is produced, stored, and processed. These steps can also help in establishing disease management strategies and minimize the yield losses.

In conclusion, the present study reports the morphological and molecular characterization, pathogenicity, and toxicological potential of *A. alternata* isolates associated with post-harvest fruit rot of sweet cherry in China. The present study concludes that *A. alternata* is an important pathogenic species causing severe post-harvest sweet cherry fruit rot, and environmental factors also have a key role in disease progression. This study also highlights that another aspect is the toxigenic potential of *A. alternata* isolates associated with the sweet cherry fruit rot disease, which could be helpful for the practical implications in the cherry juice industry for the regulation limits of *Alternaria* mycotoxins in cherry-based foods. The actual risk and level of contamination of cherry juice by *Alternaria* mycotoxins during the industrial processing have not yet been quantified clearly and need to be investigated. Therefore, the precise identification, characterization, and toxicological potential of the causal pathogen are necessary for the proper management of the disease and for understanding the pathogen behavior including virulence and mode of infection. Characterization of *A. alternata* is crucial to minimize losses caused by cherry fruit rot. Ingesting fruits contaminated with *A. alternata* and their mycotoxins can lead to health problems, posing a public health risk. Furthermore, the determination of the disease incidence, fungal diversity, and characterization of causal agents in other provinces of China are a necessary task to reduce the cherry fruit losses. Early pathogen detection and characterization can be helpful in effectively managing cherry fruit rot disease and may provide timely insights into the disease’s progression.

## Data availability statement

The datasets presented in this study can be found in online repositories. The names of the repository/repositories and accession number(s) can be found in the article/Supplementary material.

## Author contributions

TA: Conceptualization, Formal analysis, Methodology, Validation, Visualization, Writing – original draft. FX: Writing – review & editing. CC: Writing – review & editing. YL: Conceptualization, Funding acquisition, Investigation, Project administration, Supervision, Writing – review & editing.

## Footnotes

^1^ http://www.leica-microsystems.com
