# Supplementary material for: Characterization and toxicological potential of Alternaria alternata associated with post-harvest fruit rot of Prunus avium in China
Source: Front Microbiol. 2024 Feb 6;15:1273076. doi: 10.3389/fmicb.2024.1273076 (PMC10877066; doi:10.3389/fmicb.2024.1273076)
Supplement: Supplementary file 1 [file Data_Sheet_1.docx]

**Table S1:** GenBank accession numbers of the representative isolates obtained in this study and reference isolates from NCBI used for multi-gene phylogenetic analyses.

| **Species** | **Location** | **Host/substrate** | **Isolate code*** | **ITS** | **Endo-PG** | **Alt a1** |
| --- | --- | --- | --- | --- | --- | --- |
| *A. alternata* | China | *Prunus avium* | **ACT-2** | MN548781 | MN575702 | MN575708 |
|  | China | *P. avium* | **ACT-3** | MN548782 | MN575703 | MN636865 |
|  | China | *P. avium* | **ACT-4** | MN548783 | MN575704 | MN636866 |
|  | China | *P. avium* | **ACT-5** | MN548784 | MN575705 | MN636867 |
|  | China | *P. avium* | **ACT-6** | MN548785 | MN575706 | MN636868 |
|  | China | *Broussonetia papyrifera* | CBS 121455 | KP124368 | KP123916 | KP124072 |
|  | China | *Sanguisorba officinalis* | CBS 121456 | KP124369 | KP124073 | KP123917 |
|  | China | *Platycodon grandiflorus* | CBS 121348 | KP124367 | KP124070 | KP123915 |
|  | South Africa | *Malus domestica* | CBS 113013 | KP124341 | KP124042 | KP123889 |
|  | Sahara Desert | desert sand | CBS 686.68 | KP124306 | KP124006 | KP123859 |
|  | Japan | *Impatiens hawkeri* | MUCC 1610 | LC269968 | LC276242 | LC276230 |
|  | Japan | *Pelargonium hortorum* | MUCC 1616 | LC269969 | LC276243 | LC276231 |
|  | USA | *Staphylea trifolia* | CBS 154.31 | KP124301 | KP123998 | KP123851 |
|  | USA | *Citrus reticulata* | CBS 102600 | KP124331 | KP124033 | KP123880 |
|  | Canada | Human arm tissue | CBS 109455 | KP124335 | KP124036 | KP123883 |
|  | Egypt, | Soil | CBS 103.33 | KP124302 | KP123999 | KP123852 |
|  | Denmark, | *Godetia* sp. | CBS 117.44 | KP124303 | KP124001 | KP123854 |
|  | Germany | *Lolium* sp. | CBS 826.68 | KP124307 | KP124007 | KP123860 |
|  | Kuwait | Soil | CBS 198.74 | KP124310 | KP124010 | KP123863 |
|  | Greece | *Prunus* sp. | CBS 119115 | KP124360 | KP124062 | KP123909 |
|  | Israel | *Minneola tangelo* | CBS 121344 | KP124365 | KP124068 | KP123913 |
|  | South Africa | *Minneola tangelo* | CBS 121346 | KP124366 | KP124069 | KP123914 |
| *A. betae-kenyensis* | Kenya | *Beta vulgaris* var. *cicla* | CBS 118810^T^ | KP124419 | KP124123 | KP123966 |
| *A. eichhorniae* | India | *Eichhornia crassipes* | CBS 489.92^T^ | KC146356 | KP124130 | KP123973 |
| *A. gaisen* | Japan | Fragaria×ananassaHS-138’ | MAFF 242310 | LC269973 | LC276252 | LC276237 |
| *A. iridiaustralis* | Australia | *Iris* sp. | CBS 118486^T^ | KP124435 | KP124140 | KP123981 |
| *A. geophila* | Switzerland | peat soil | CBS 101.13 | KP124392 | KP124096 | KP123940 |
| *A. alstroemeriae* | USA | *Alstroemeria* sp. | CBS 118808 | KP124296 | KP123993 | KP123845 |
| *A. longipes* | USA | *Nicotiana tabacum* | CBS 121333 | KP124444 | KP124150 | KP123990 |
| *A. gaisen* | Portugal | unknown | CPC 25268 | KP124428 | KP124133 | KP123976 |
| *A. arborescens* | South Africa | *Malus domestica* | CBS 115516 | KP124403 | KP124107 | KP123950 |
| *A. burnsii* | India | *Cuminum cyminum* | CBS 107.38 | KP124420 | KP124124 | KP123967 |
|  | United Kingdom | *Sorghum* sp. | CBS 879.95 | KP124422 | KP124126 | KP123969 |
| *A. jacinthicola* | Mauritius | *Arachis hypogaea* | CBS 878.95 | KP124437 | KP124142 | KP123983 |
| *A. alternantherae* | China | *Solanum melongena* | CBS 124392 | KC584179 | - | KP123846 |

*The representative isolates from this study are bold highlighted.

**Table S2.** Different growth media used in this study.

| Sr# | Growth medium name | Abbreviation | Manufactures |
| --- | --- | --- | --- |
| 1 | Potato dextrose agar | PDA | Beijing Land bridge |
| 2 | Malt extract agar | MAE | Beijing Aobox bio tech |
| 3 | Martin agar medium modified | MAM modified | Hope bio-tech |
| 4 | Dichloran-glycerol (DG-18) Agar base medium | DG18 | Beijing Aobox bio tech |
| 5 | Trypticase Soy Agar medium | TSA | Beijing Aobox bio tech |
| 6 | Salt czapek dox agar medium | SCDA | Beijing Aobox bio tech |
| 7 | Dichloran Rose Bengal Chloramphenicol Agar | DRBC | Hope bio-tech |
| 8 | De Man Rogosa and Sharpe agar | MRS | Beijing Land bridge |
| 9 | *Aspergillus flavus* and *parasiticus* agar | AFPA | Hope bio-tech |


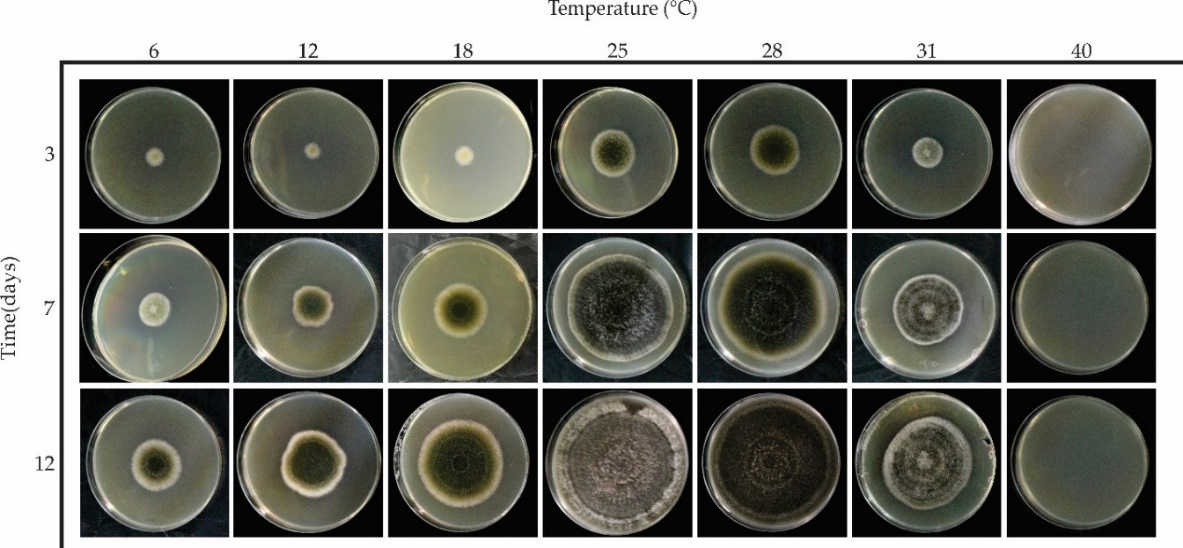


**Figure S1.** Growth of isolate ACT-3 at different temperatures on PDA medium at 3, 7, and 12 dpi.


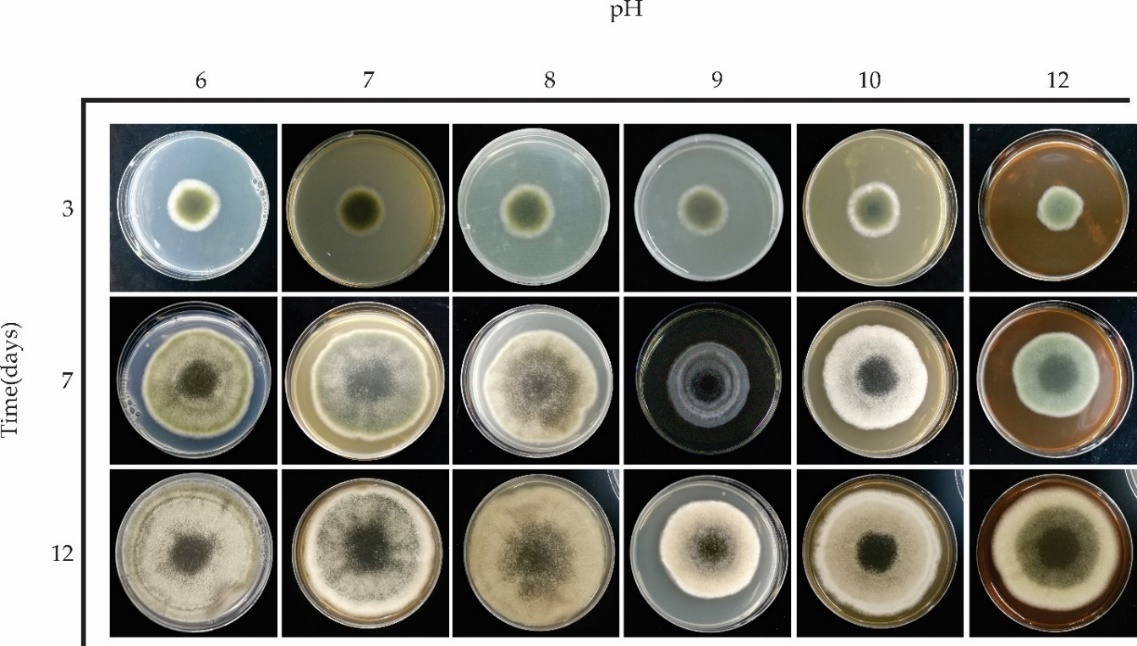


**Figure S2.** Color variations of *A. alternata* isolate ACT-3 at different pH levels at 3, 7, and 12 dpi on PDA medium.


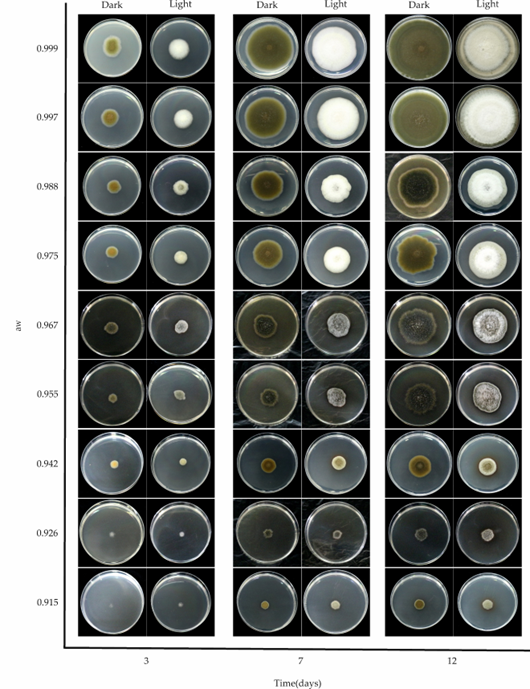


**Figure S3.** *A. alternata* isolate ACT-3 showed different colony colors on different levels of a_w_ at 3, 7, and 12 dpi on PDA and 28 ⁰C. Dark= 24 hours dark, Light= 24 hours light (LED) exposure.

**1S: Effect of different media on fungal growth:**

*Alternaria alternata* showed the potential to grow well on all types of culture media tested in this study. Among the different media tested, the MAM modified showed the highest mean mycelial growth of 84 mm followed by PDA (83 mm), DG18 (83 mm), and MAE (70 mm). The least mean mycelial growth was observed on MRS (35 mm) followed by SCD (45 mm) and TSA (50 mm) at 28 °C with 24h darkness at 12 dpi (**Figure S4a**). *A. alternata* showed light green, olive and light brown colonies with white aerial mycelium at the colony edges at 3, 7 and 12 dpi respectively in 24 h darkness at 28 ⁰C on PDA. On MEA, ACT-3 showed whitish growth with a light brown ring at the center of the colony, greenish to olive, and off-white with greenish to brown at the center at 3, 7, and 12 dpi respectively in 24 darkness at 28 ⁰C. ACT-3 showed light golden, greenish to light golden, and light brown to olive colonies at 3, 7, and 12 dpi respectively on MAM modified and DG18, while on TSA, MRS, and AFPA agar medium, the fungal colonies appeared olive to dark green with light brownish shade and colony growth was slightly irregular in 24 h darkness. ACT-3 mycelium spread very clearly, making thick branches and producing olive color after 7 dpi on SCD agar medium in 24 h darkness while on DRBC agar fungal colony showed light brown, light brownish to olive and olive colony with white aerial hyphae when incubated under dark condition. When incubated at 24 h light, the highest colony growth was 82 mm on MAM and PDA. The lowest mycelial growth was 29 mm on (**Figure S4 b**). Whereas isolate ACT-3 showed off white to light creamy colored and cottony to the velvety colony at 3, 7, and 12 dpi on all types of tested media when incubated under 24h light condition (white light LED) at 28 °C (**Figure S5**). The results revealed that the best media for the growth of *A. alternata* are MAM, PDA, and DG18 in both dark and light incubation conditions.


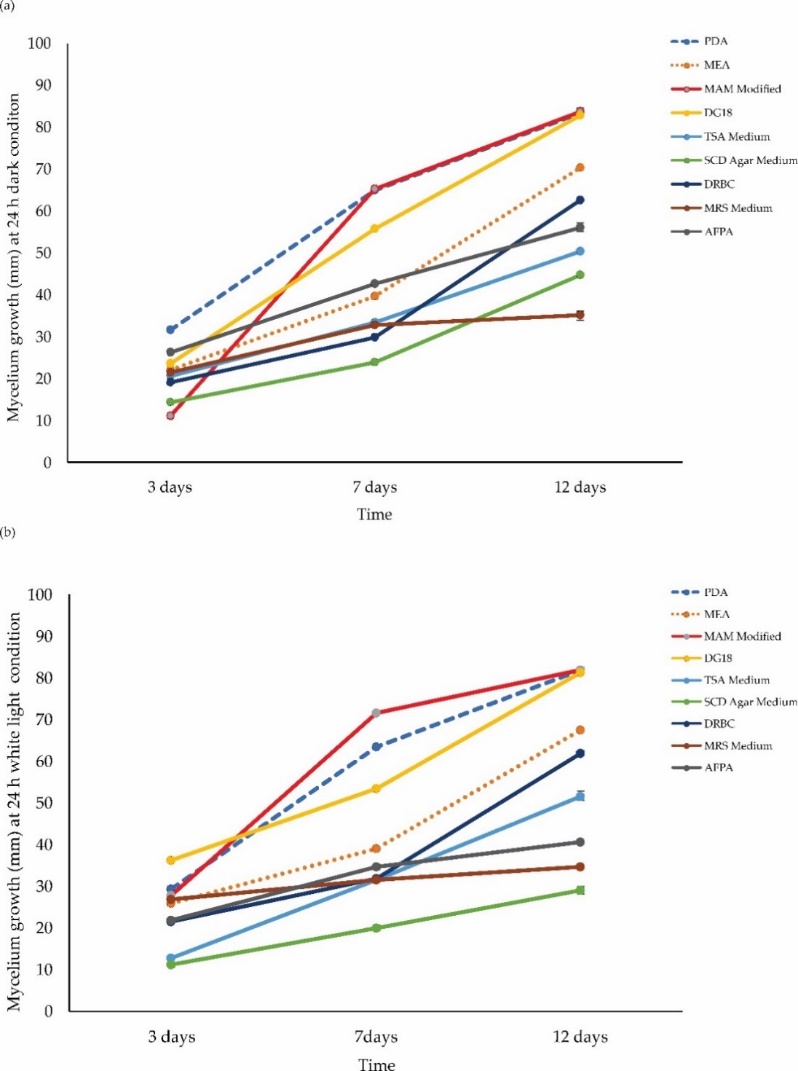


**Figure S4.** Effect of different culture media on the growth of *A. alternata* isolate ACT-3 (a) Mycelial growth under 24 h dark on tested media (b) Mycelial growth under 24 h light (LED) exposure on tested media. Bars indicate the SE of mean values.


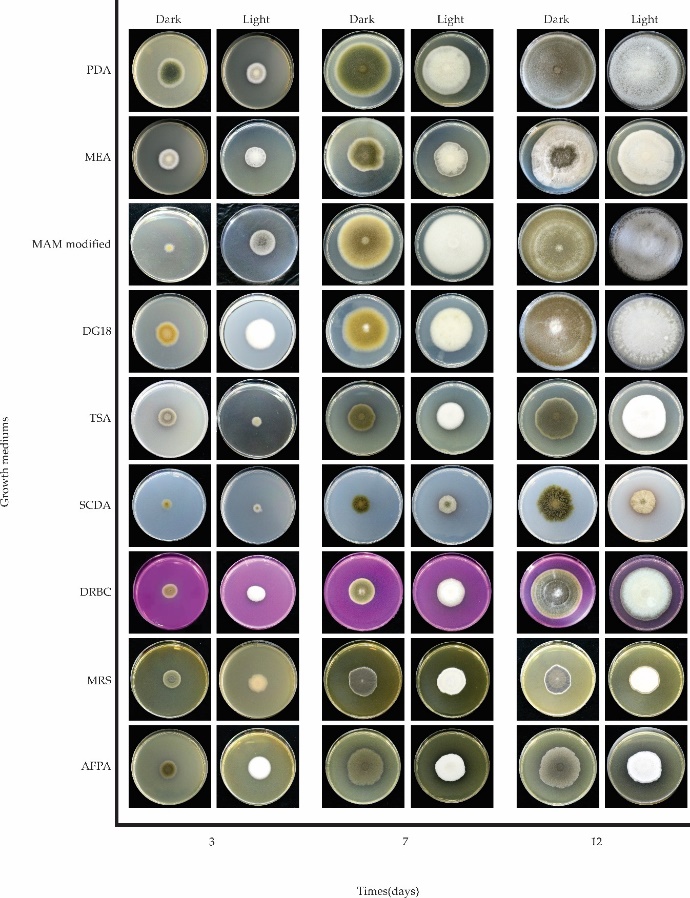


**Figure S5.** *A. alternata* isolate ACT-3 showed different colony colors on selected growth media at 3, 7, and 12 dpi. Dark= 24 hours darkness, Light= 24 hours white light (LED) exposure.


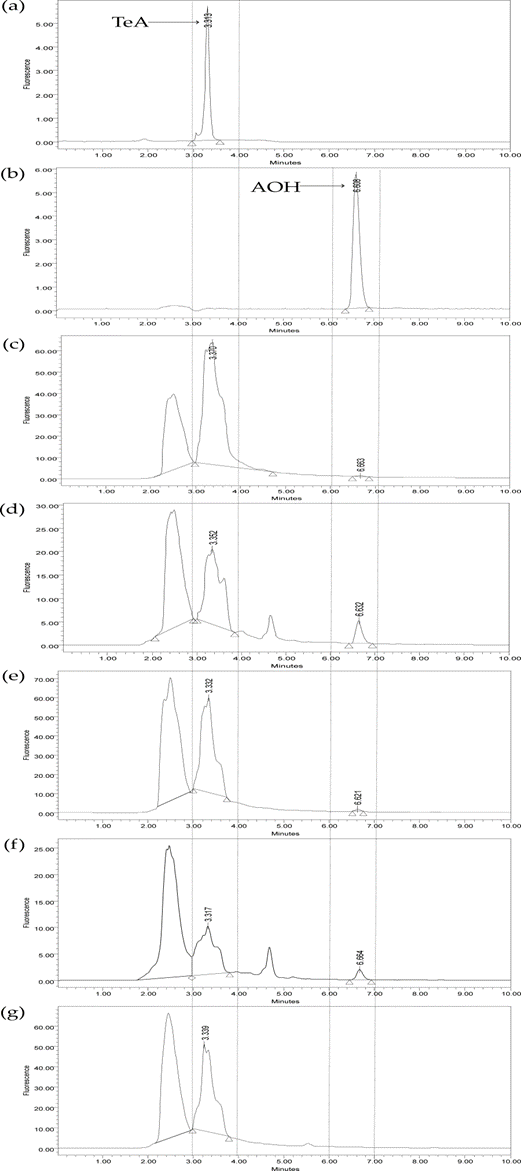


**Figure S6.** HPLC chromatograms of TeA and AOH recoded from toxigenic isolates of *A. alternata* (a) standard chromatogram for tenuazonic acid (b) standard chromatogram for alternariol (c) chromatogram for ACT-2 (d) chromatogram for ACT-3 (e)chromatogram for ACT-4 (f) chromatogram for ACT-5 and (g) chromatogram for ACT-6.
